# Supplementary figures and images for: EXOSC10 is a novel hepatocellular carcinoma prognostic biomarker: a comprehensive bioinformatics analysis and experiment verification
Source: PeerJ. 2023 Sep 8;11:e15860. doi: 10.7717/peerj.15860 (PMC10494838; doi:10.7717/peerj.15860)

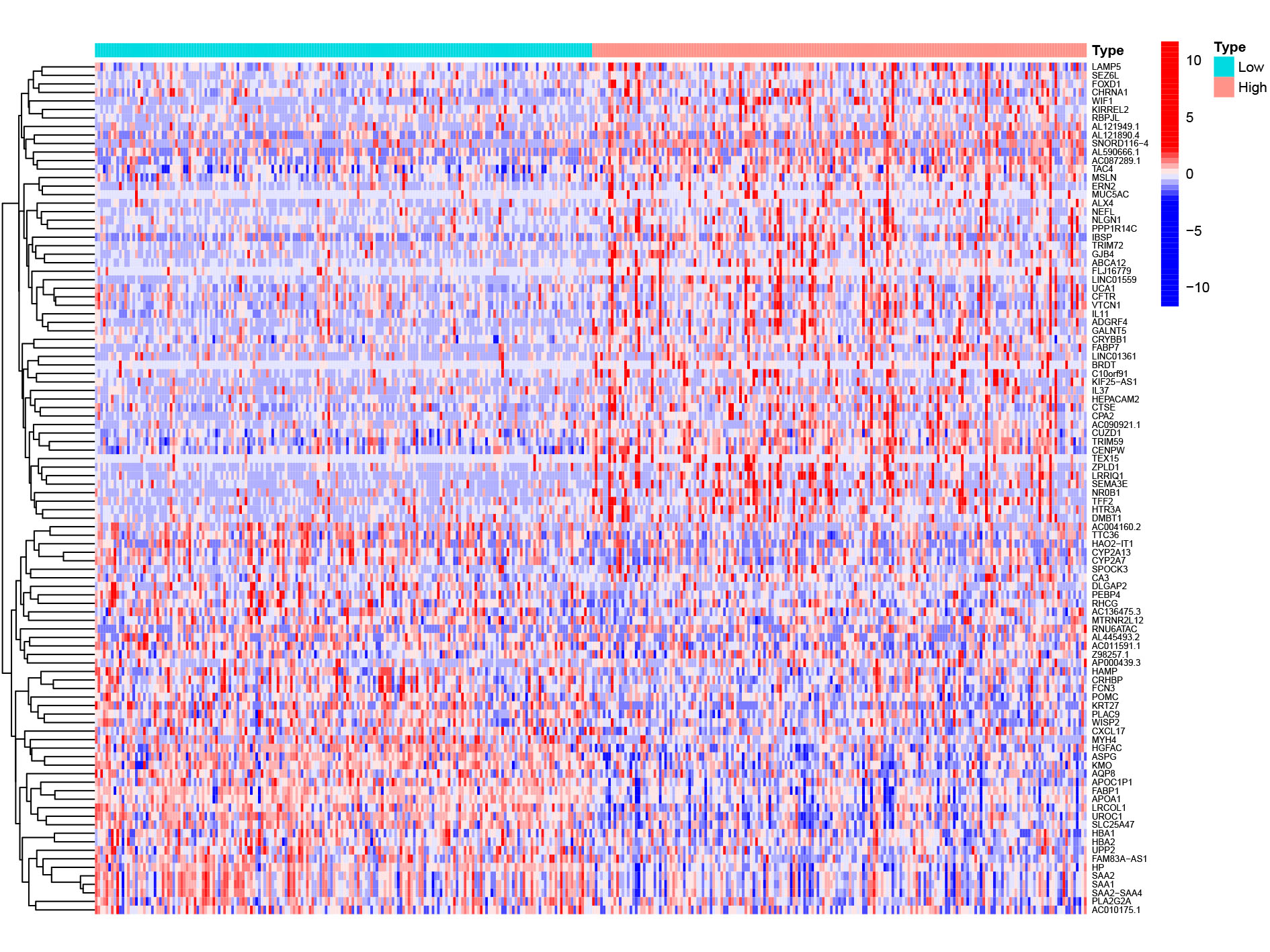

Supplement: Supplemental Information 1 [file peerj-11-15860-s001.jpg]

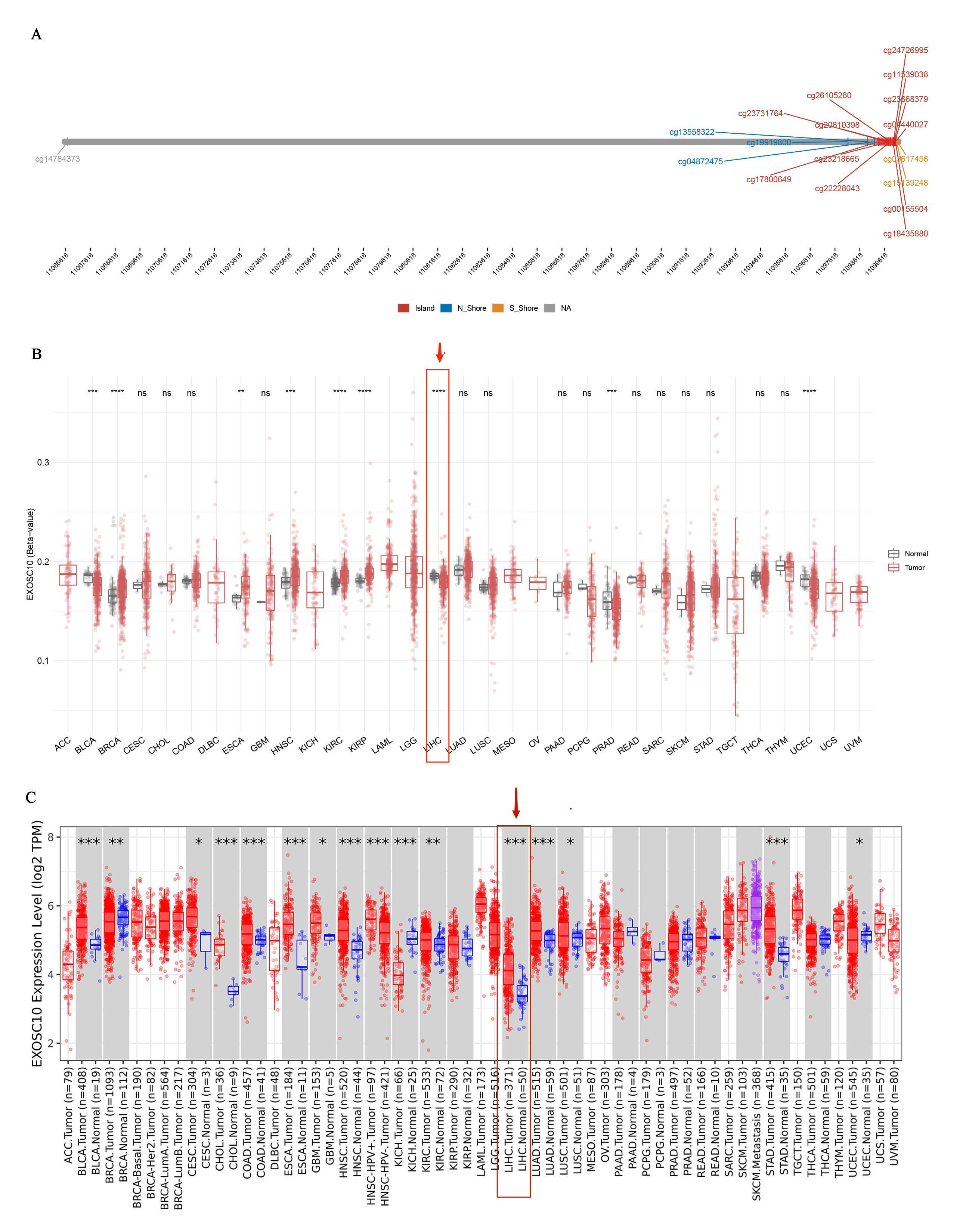

Supplement: Supplemental Information 2 — (A) Distribution of EXOSC10 methylation sites in various tumor types. (B) Pan-tumor analysis of EXOSC10 methylation in tumor and normal tissues. (C). The expression of EXOSC10 in different tumor types in TIMER. Note: ns: p > 0.05; *: p < = 0.05; **: p < = 0.01; ***: p < = 0.001; ****: p < = 0.0001. [file peerj-11-15860-s002.jpg]

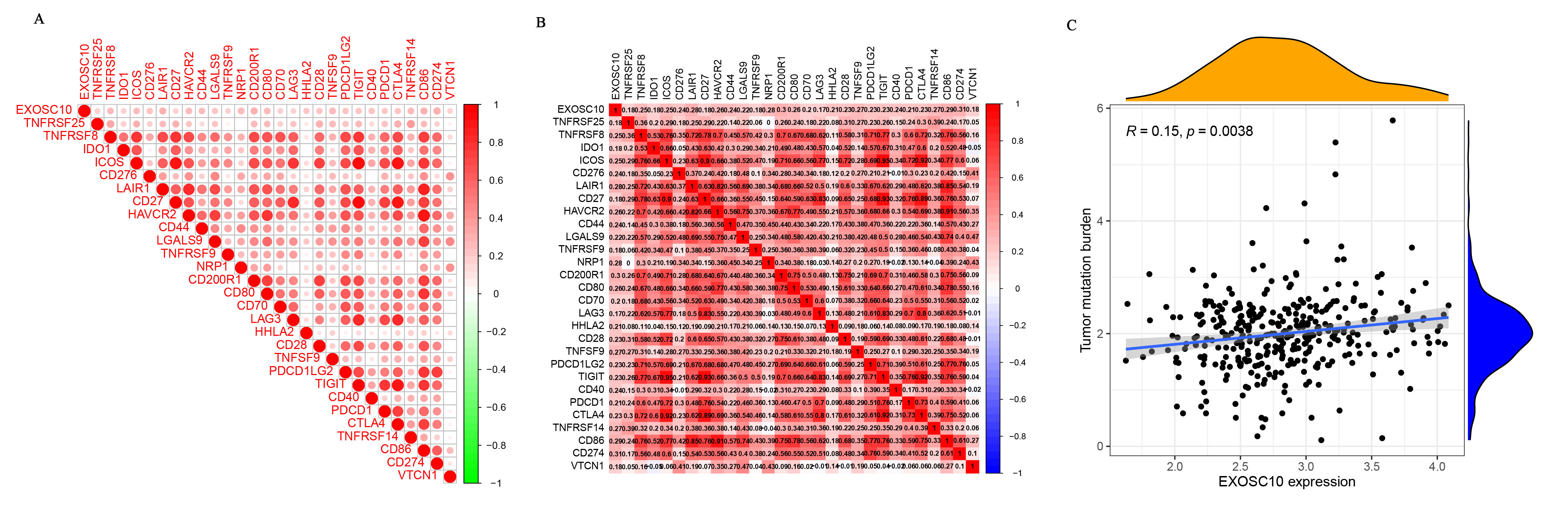

Supplement: Supplemental Information 3 — (A–B) The association between EXOSC10 and HCC-related checkpoints. (C) The association between EXOSC10 and TMB in HCC. [file peerj-11-15860-s003.jpg]

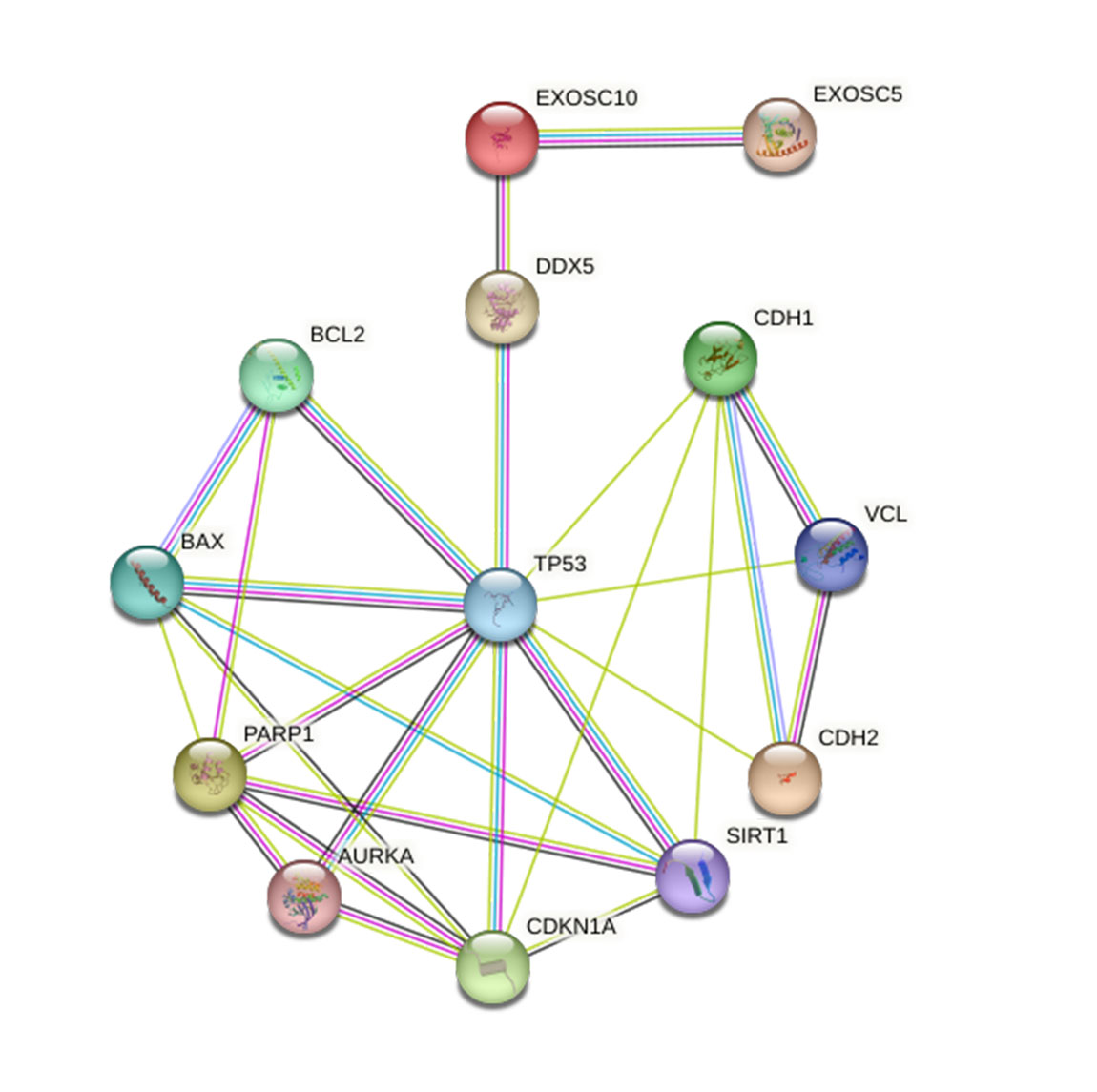

Supplement: Supplemental Information 4 — EXOSC10 may regulate p53 signaling pathway by regulating the expression of DDX5. [file peerj-11-15860-s004.jpg]

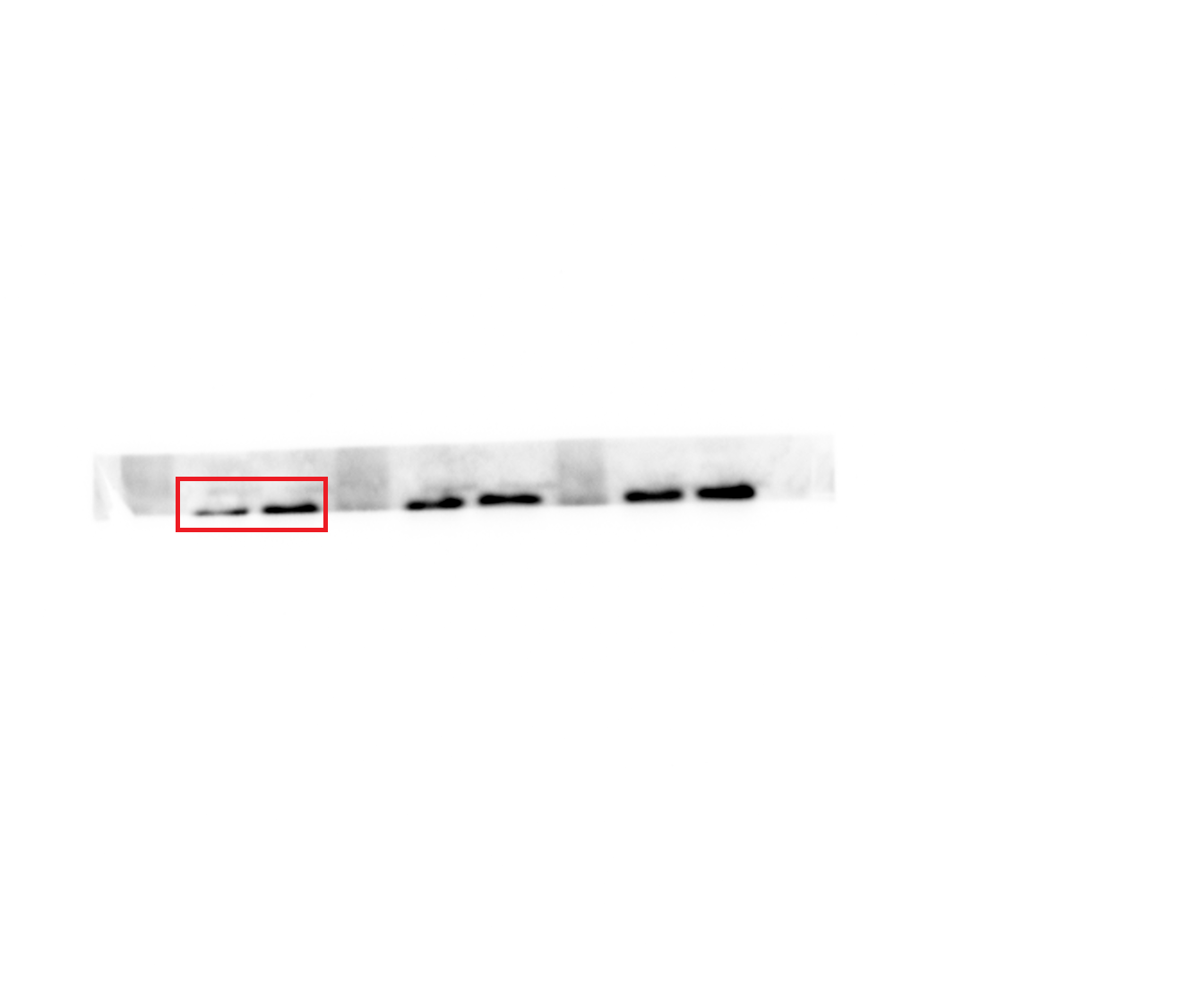

Supplement: Supplemental Information 6 [file peerj-11-15860-s006.zip › raw data-western blot/Western Blots images/Western Blot/Original Image for Fig 10A/Bax-original drawing.Tif]

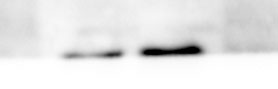

Supplement: Supplemental Information 6 [file peerj-11-15860-s006.zip › raw data-western blot/Western Blots images/Western Blot/Original Image for Fig 10A/Bax.jpg]

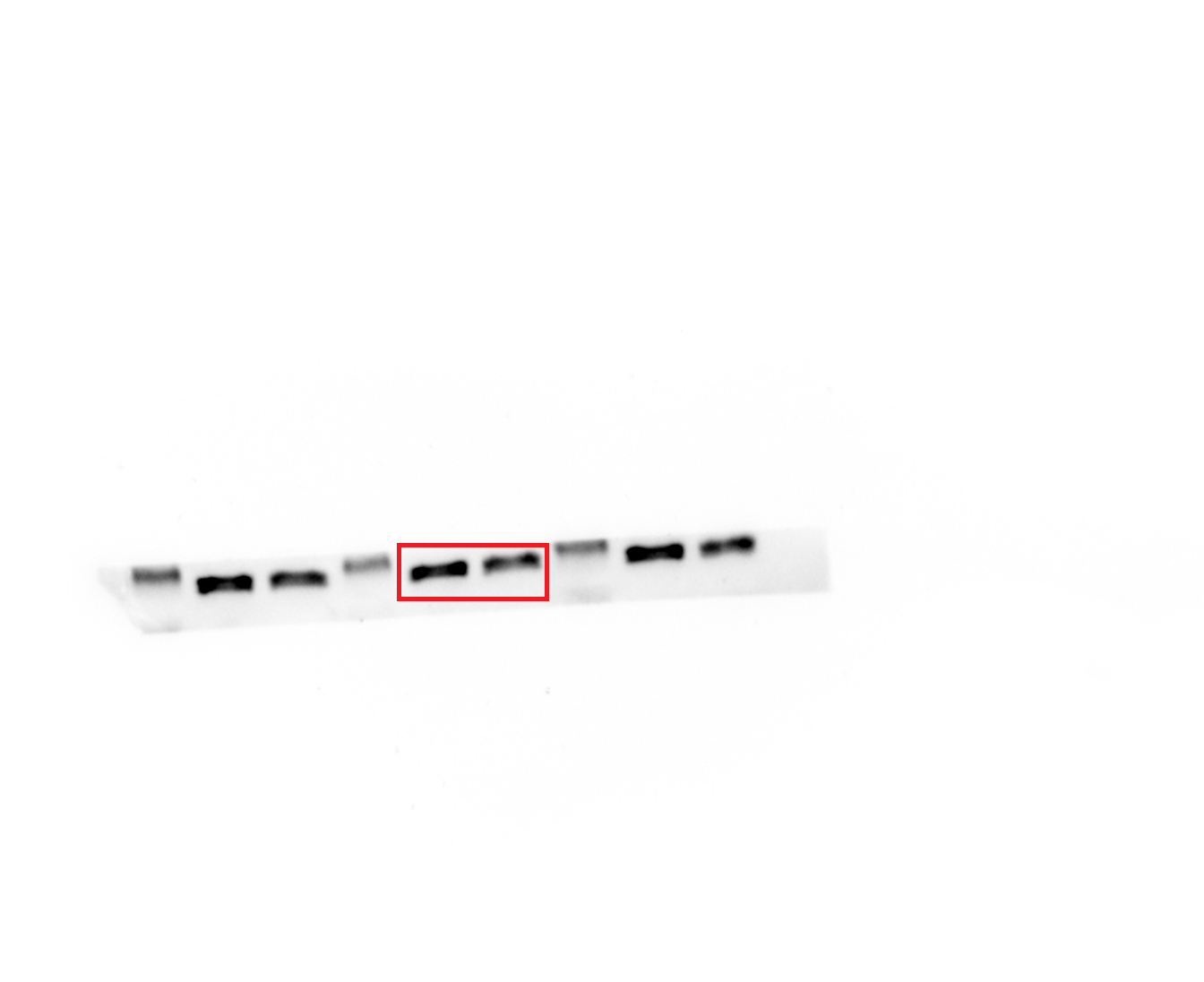

Supplement: Supplemental Information 6 [file peerj-11-15860-s006.zip › raw data-western blot/Western Blots images/Western Blot/Original Image for Fig 10A/Bcl2-original drawing.Tif]

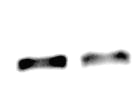

Supplement: Supplemental Information 6 [file peerj-11-15860-s006.zip › raw data-western blot/Western Blots images/Western Blot/Original Image for Fig 10A/Bcl2.jpg]

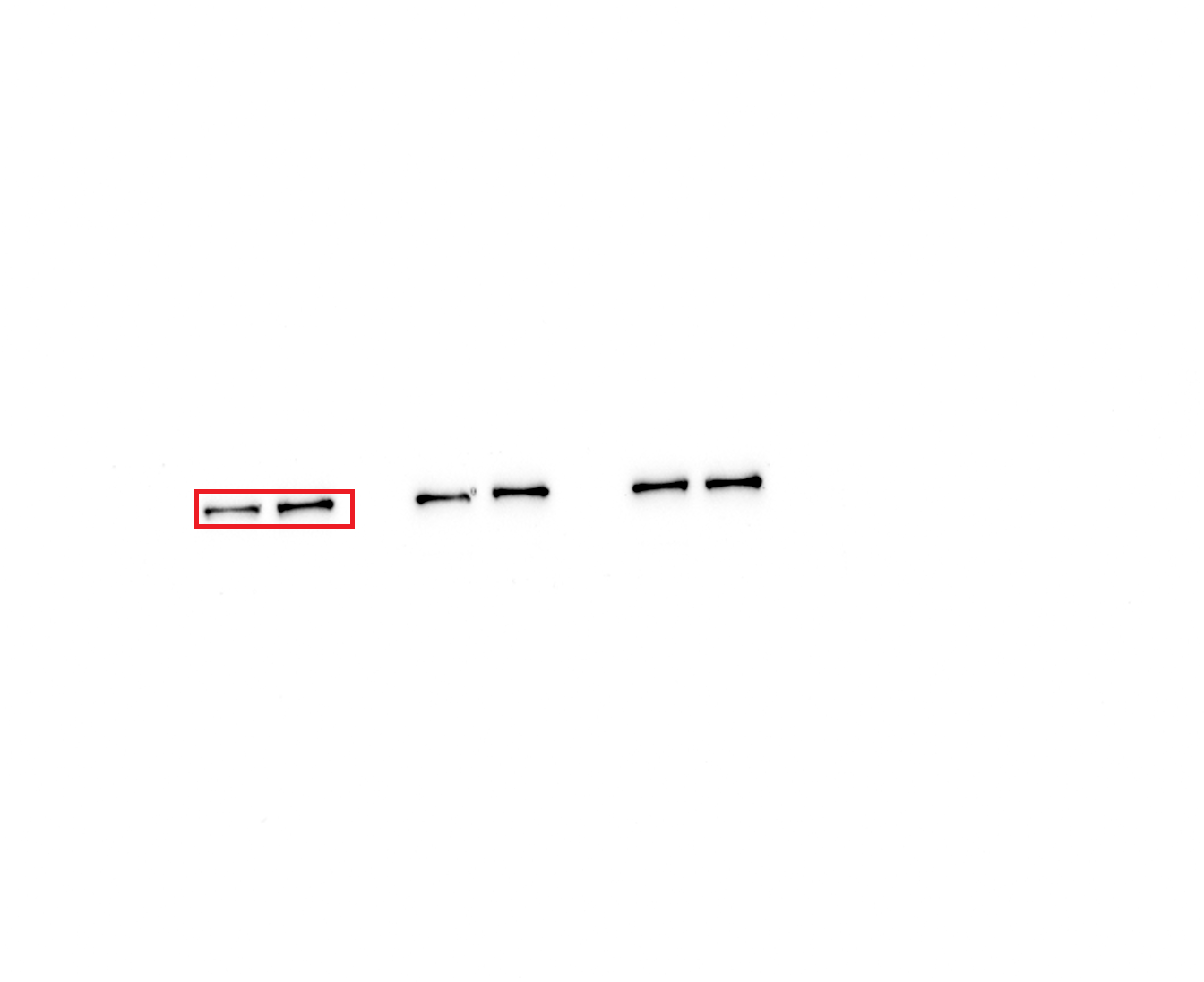

Supplement: Supplemental Information 6 [file peerj-11-15860-s006.zip › raw data-western blot/Western Blots images/Western Blot/Original Image for Fig 10A/E-cadherin-original drawing.Tif]

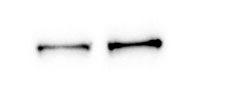

Supplement: Supplemental Information 6 [file peerj-11-15860-s006.zip › raw data-western blot/Western Blots images/Western Blot/Original Image for Fig 10A/E-cadherin.jpg]

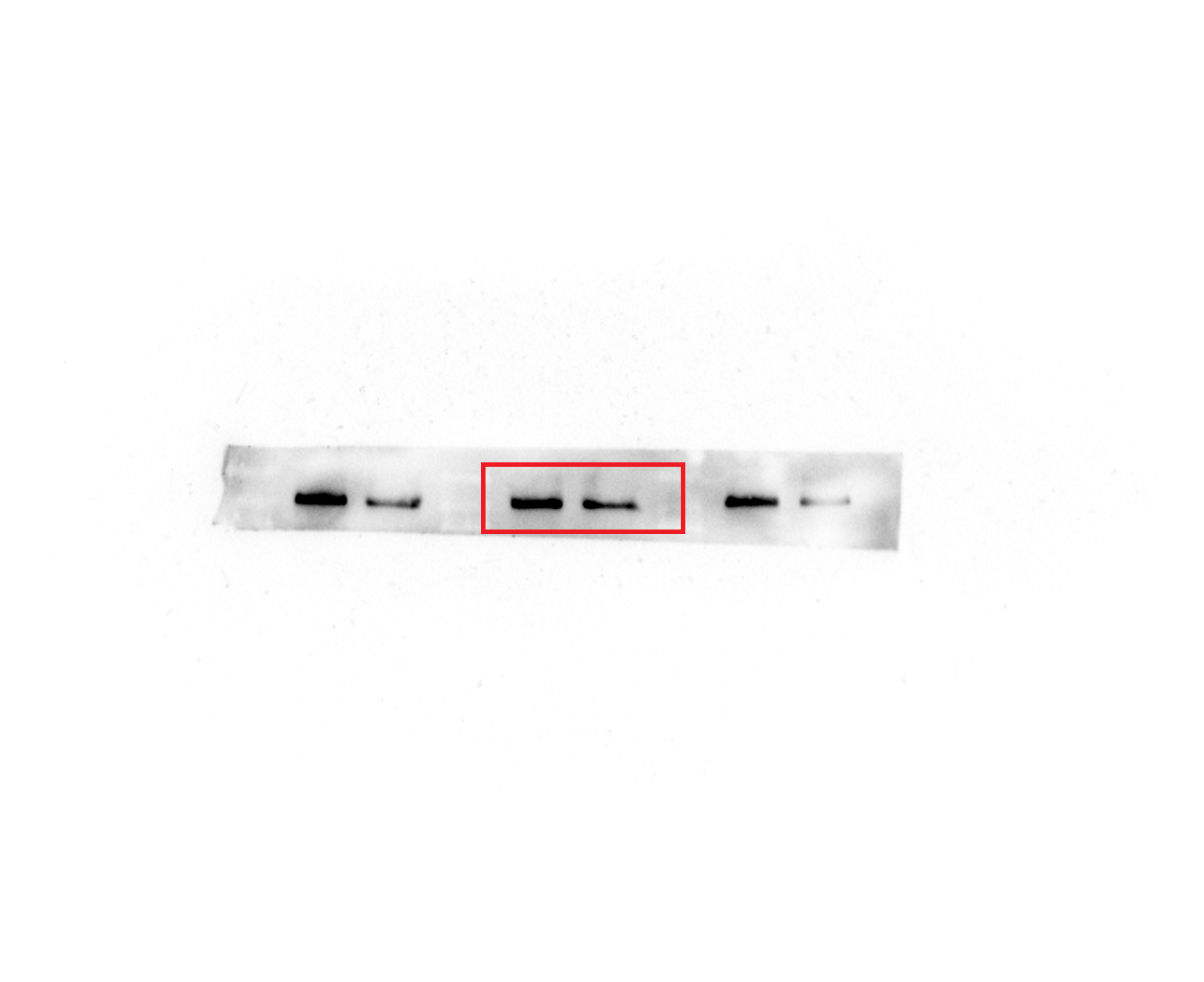

Supplement: Supplemental Information 6 [file peerj-11-15860-s006.zip › raw data-western blot/Western Blots images/Western Blot/Original Image for Fig 10A/N-cadherin-original drawing.Tif]

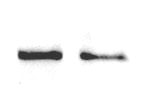

Supplement: Supplemental Information 6 [file peerj-11-15860-s006.zip › raw data-western blot/Western Blots images/Western Blot/Original Image for Fig 10A/N-cadherin.jpg]

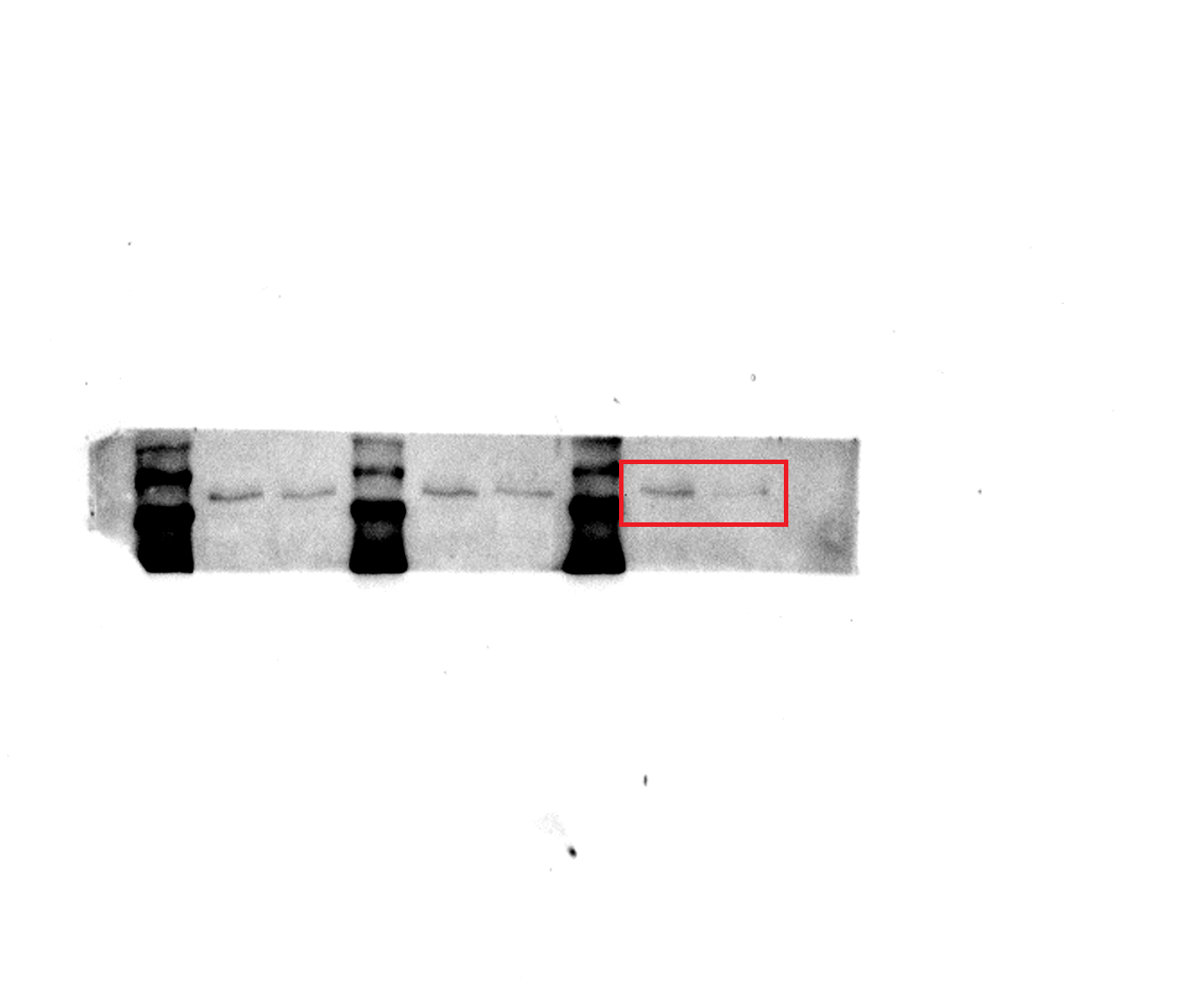

Supplement: Supplemental Information 6 [file peerj-11-15860-s006.zip › raw data-western blot/Western Blots images/Western Blot/Original Image for Fig 10A/PARP-original drawing.Tif]

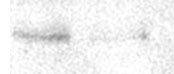

Supplement: Supplemental Information 6 [file peerj-11-15860-s006.zip › raw data-western blot/Western Blots images/Western Blot/Original Image for Fig 10A/PARP.jpg]

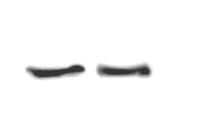

Supplement: Supplemental Information 6 [file peerj-11-15860-s006.zip › raw data-western blot/Western Blots images/Western Blot/Original Image for Fig 10A/gap.jpg]

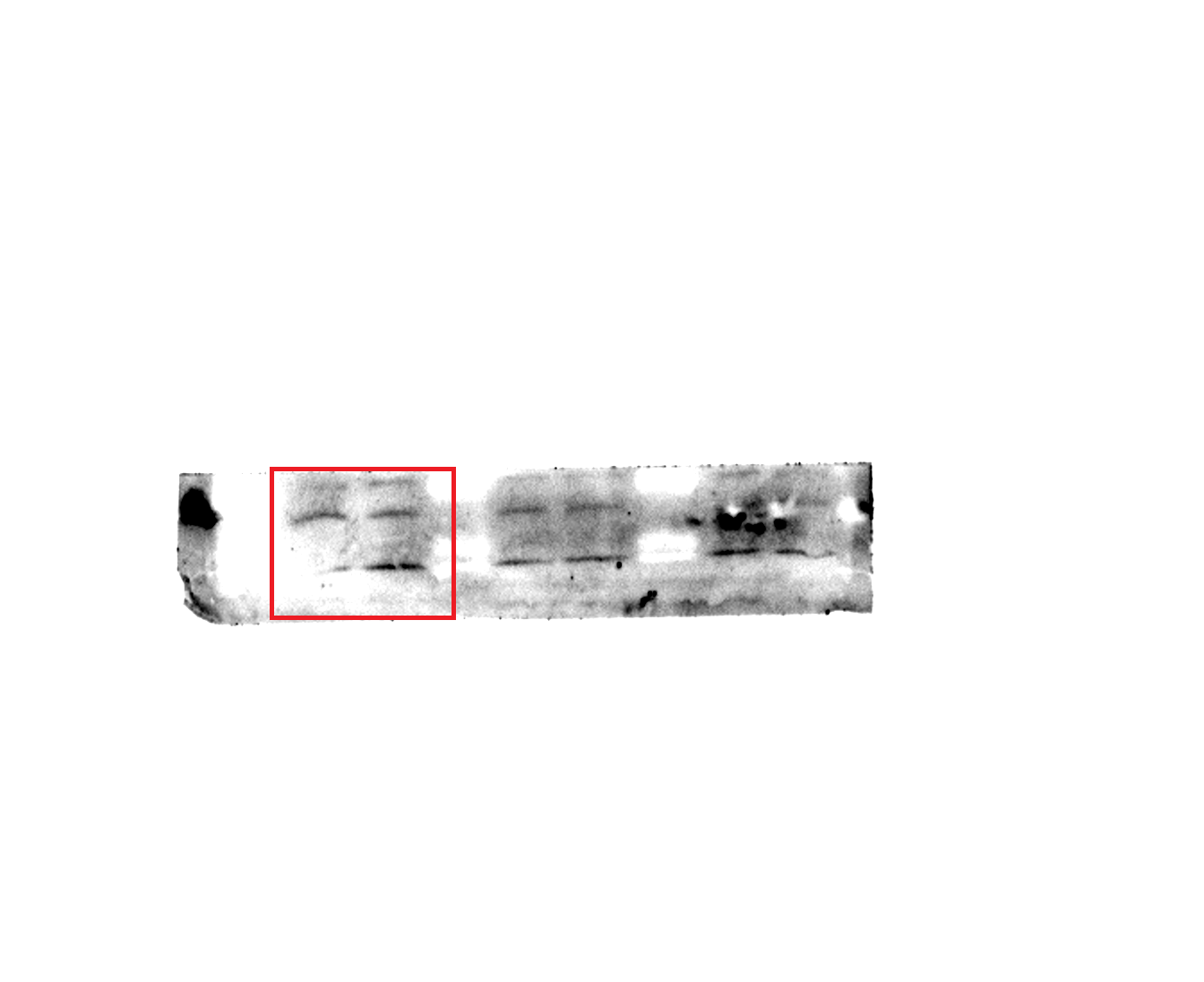

Supplement: Supplemental Information 6 [file peerj-11-15860-s006.zip › raw data-western blot/Western Blots images/Western Blot/Original Image for Fig 10A/p21-original drawing.Tif]

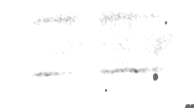

Supplement: Supplemental Information 6 [file peerj-11-15860-s006.zip › raw data-western blot/Western Blots images/Western Blot/Original Image for Fig 10A/p21.jpg]

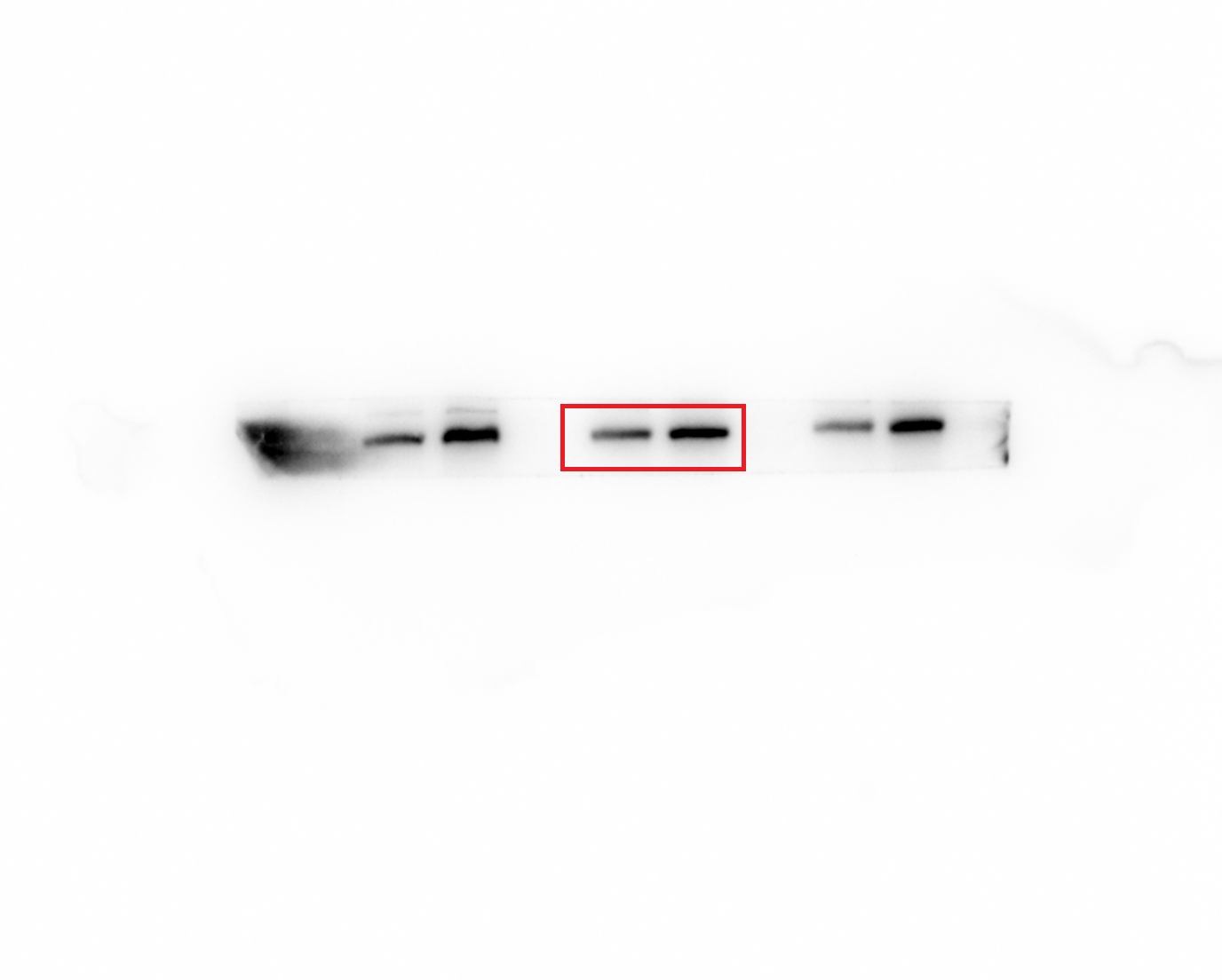

Supplement: Supplemental Information 6 [file peerj-11-15860-s006.zip › raw data-western blot/Western Blots images/Western Blot/Original Image for Fig 10A/p35-original drawing.tif]

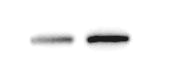

Supplement: Supplemental Information 6 [file peerj-11-15860-s006.zip › raw data-western blot/Western Blots images/Western Blot/Original Image for Fig 10A/p53.jpg]

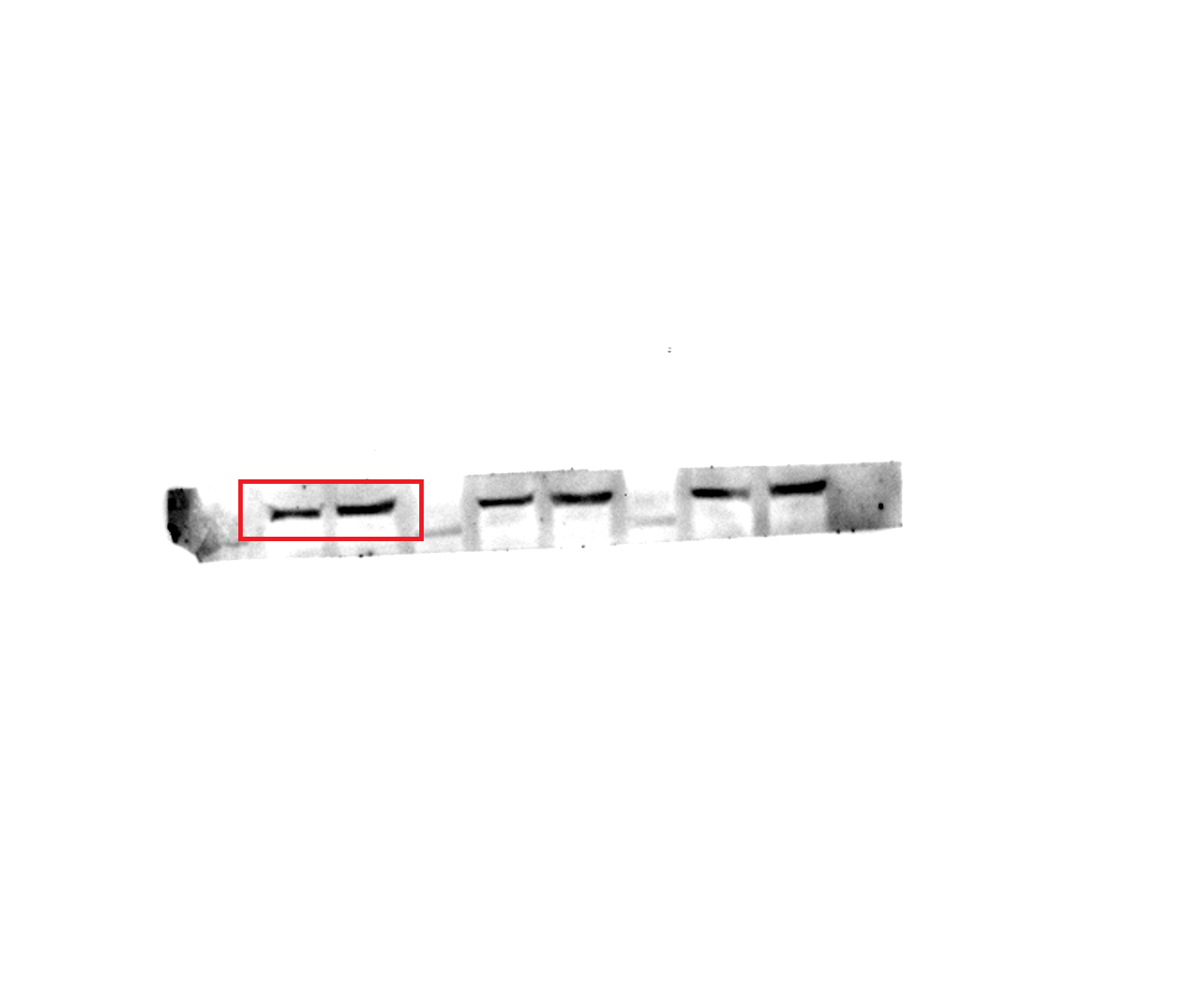

Supplement: Supplemental Information 6 [file peerj-11-15860-s006.zip › raw data-western blot/Western Blots images/Western Blot/Original Image for Fig 10A/pp53-original drawing.Tif]

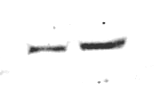

Supplement: Supplemental Information 6 [file peerj-11-15860-s006.zip › raw data-western blot/Western Blots images/Western Blot/Original Image for Fig 10A/pp53.jpg]

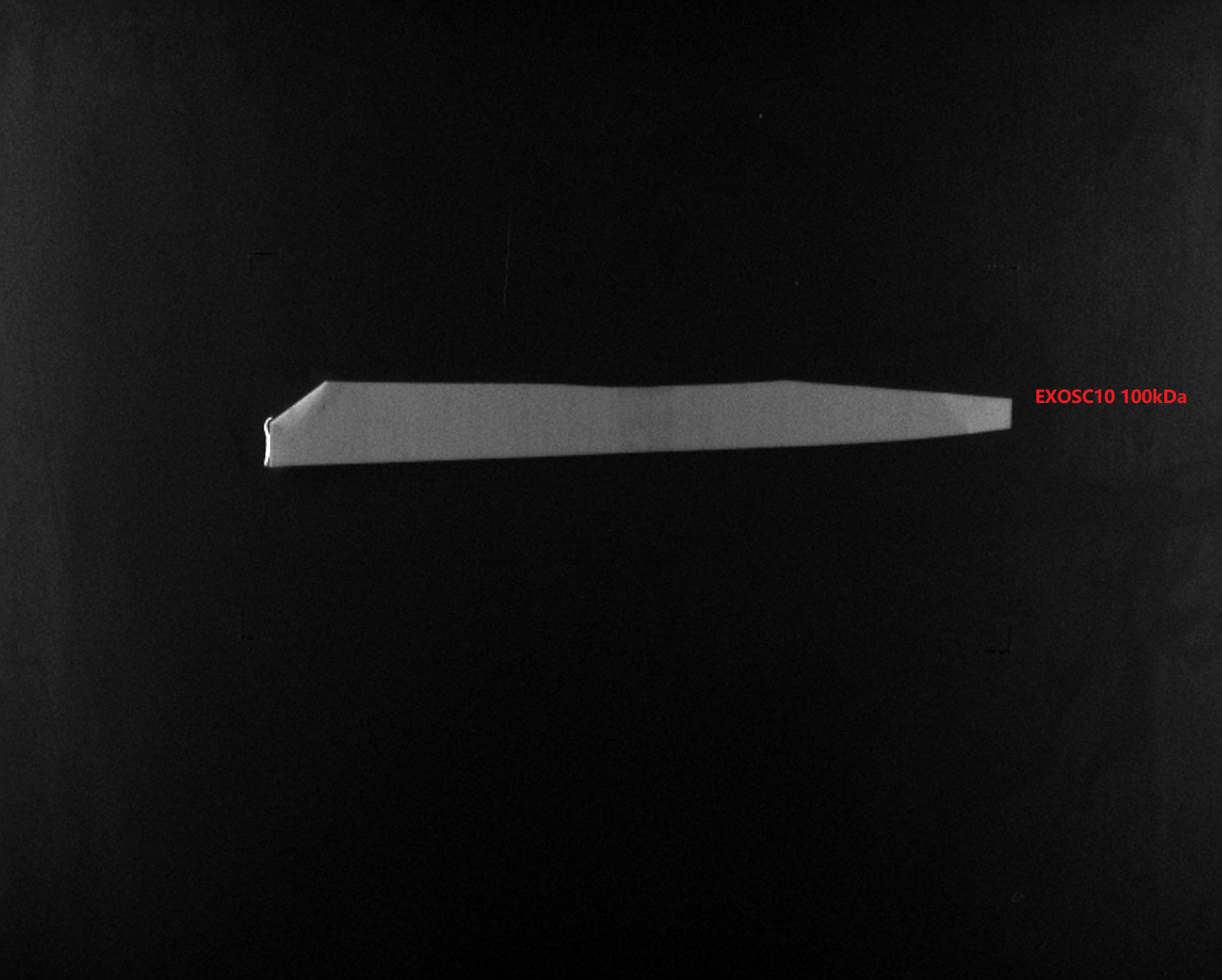

Supplement: Supplemental Information 6 [file peerj-11-15860-s006.zip › raw data-western blot/Western Blots images/Western Blot/Original Image for Fig 7A/EXOSC10-original drawing.tif]

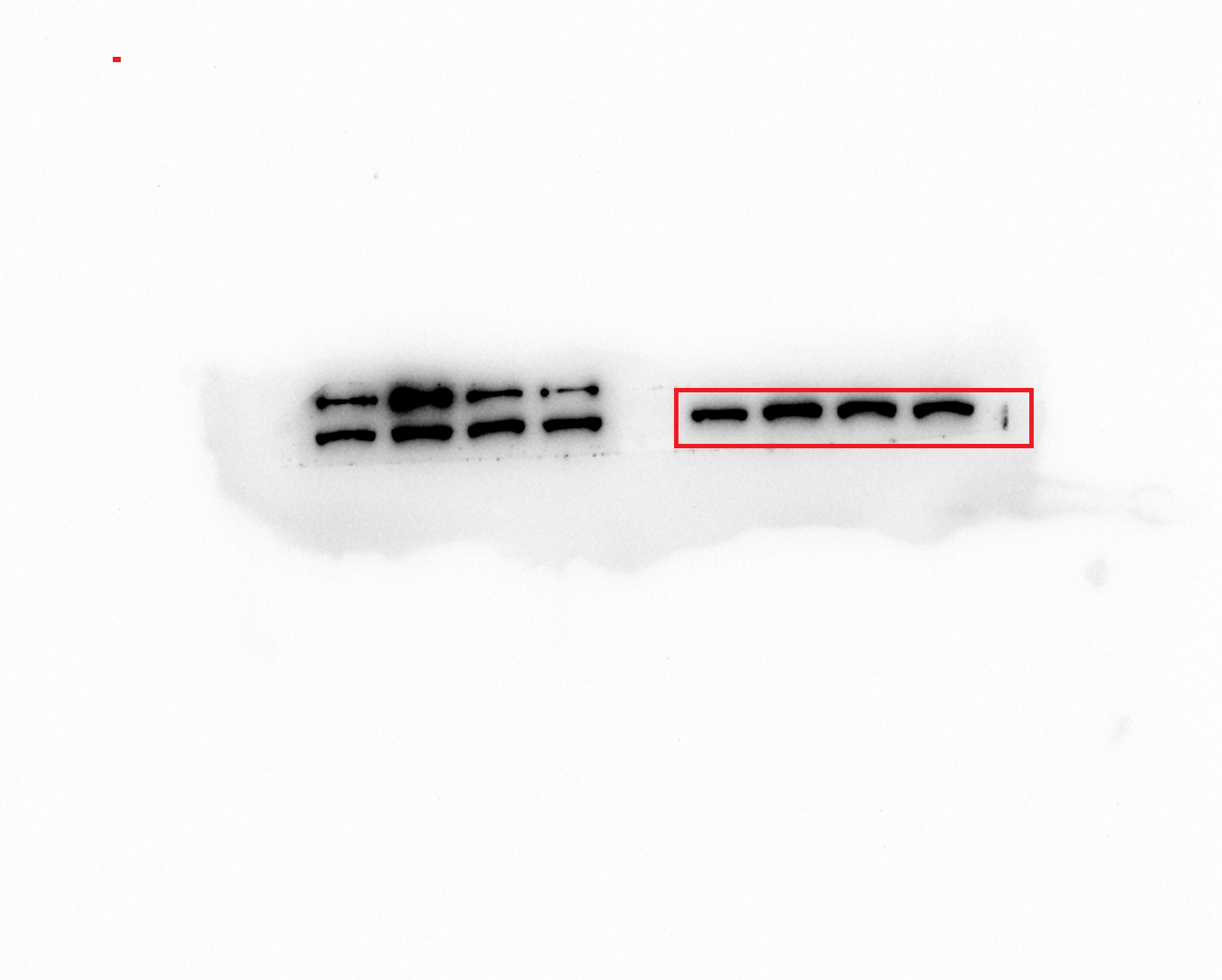

Supplement: Supplemental Information 6 [file peerj-11-15860-s006.zip › raw data-western blot/Western Blots images/Western Blot/Original Image for Fig 7A/EXOSC10.tif]

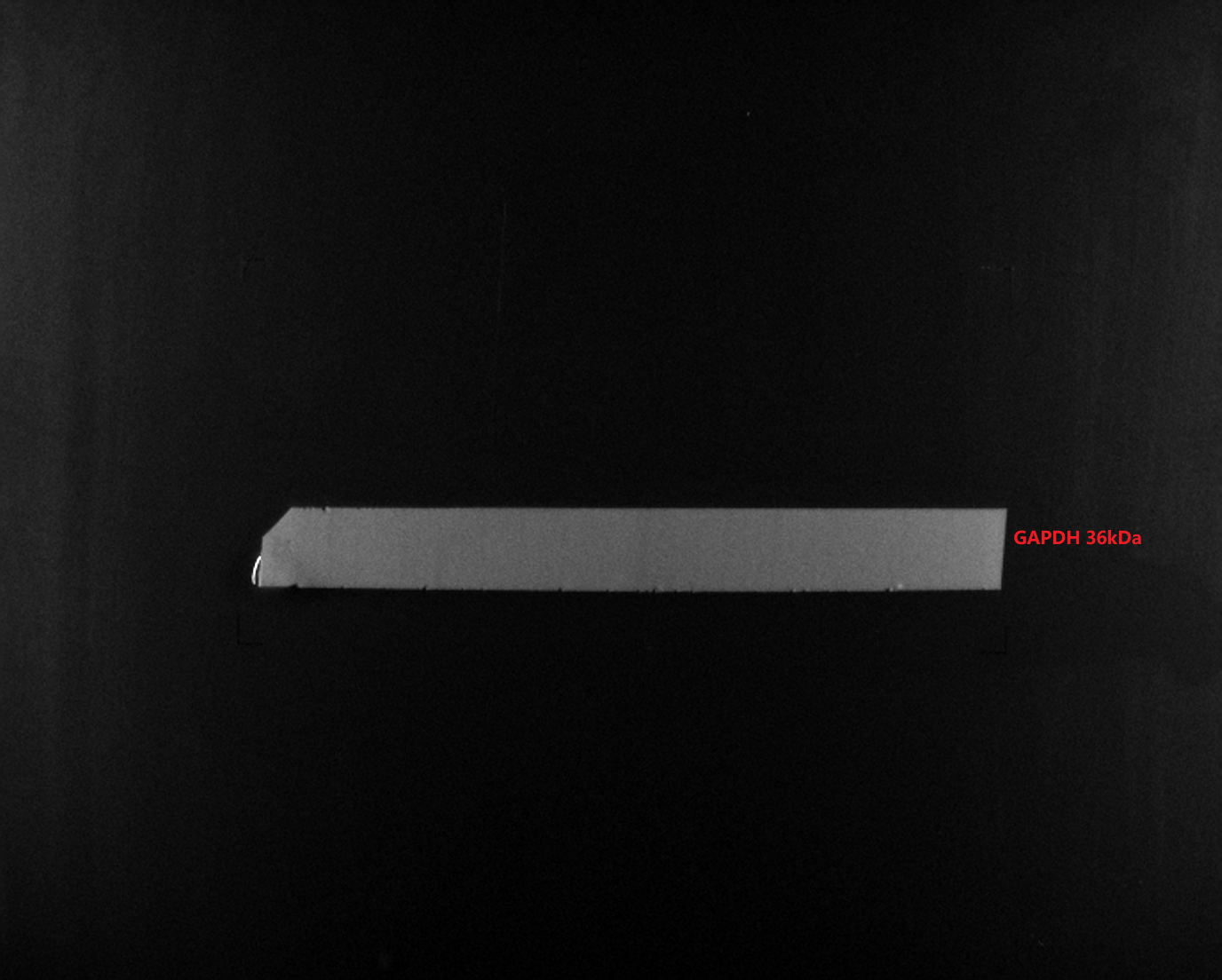

Supplement: Supplemental Information 6 [file peerj-11-15860-s006.zip › raw data-western blot/Western Blots images/Western Blot/Original Image for Fig 7A/GAPDH-original drawing.tif]

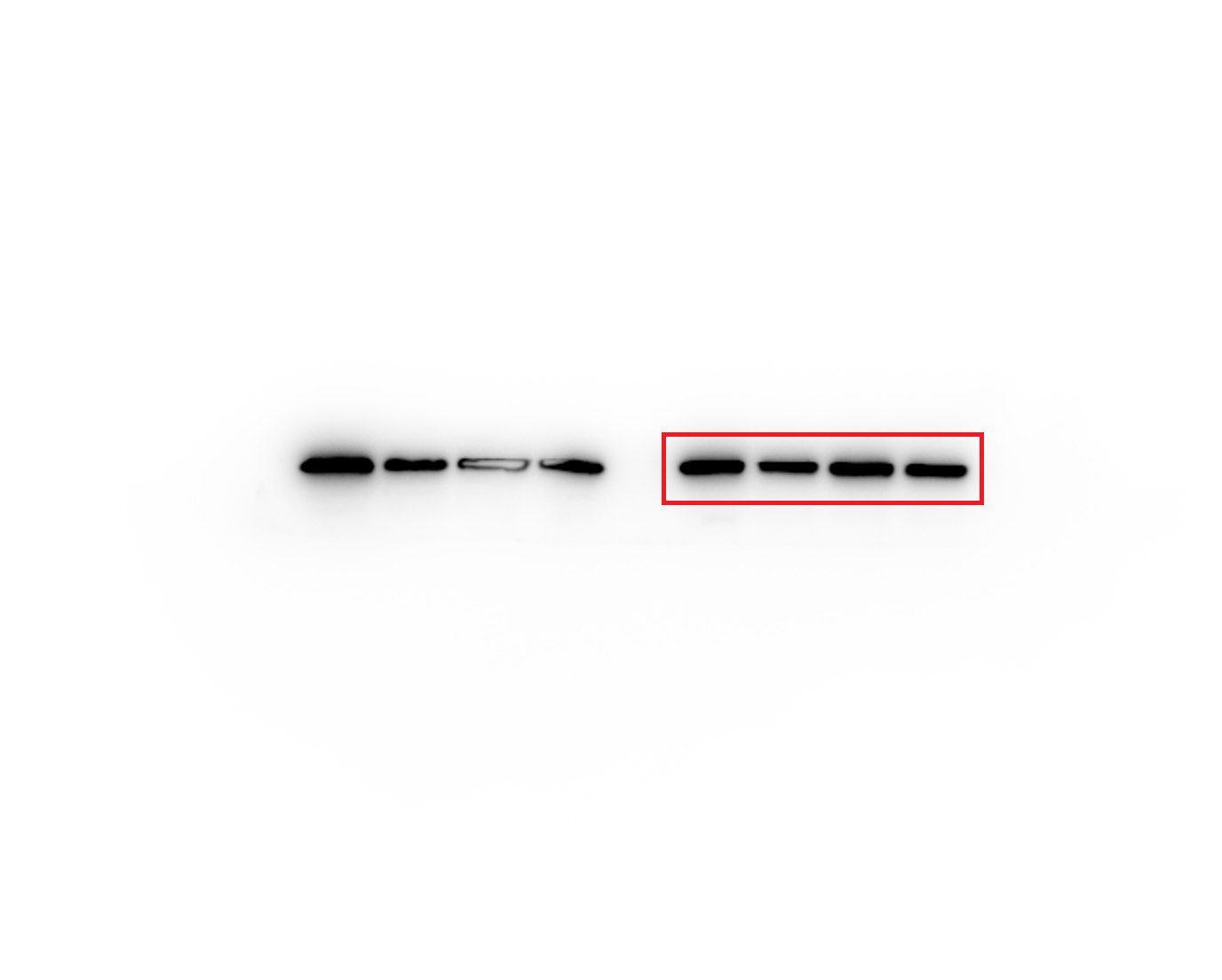

Supplement: Supplemental Information 6 [file peerj-11-15860-s006.zip › raw data-western blot/Western Blots images/Western Blot/Original Image for Fig 7A/GAPDH.tif]

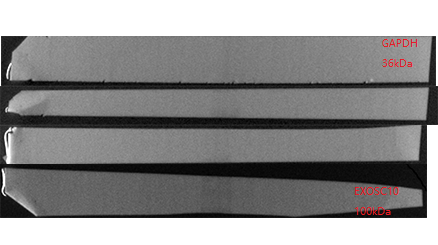

Supplement: Supplemental Information 6 [file peerj-11-15860-s006.zip › raw data-western blot/Western Blots images/Western Blot/Original Image for Fig 7A/Intact membrane.tif]

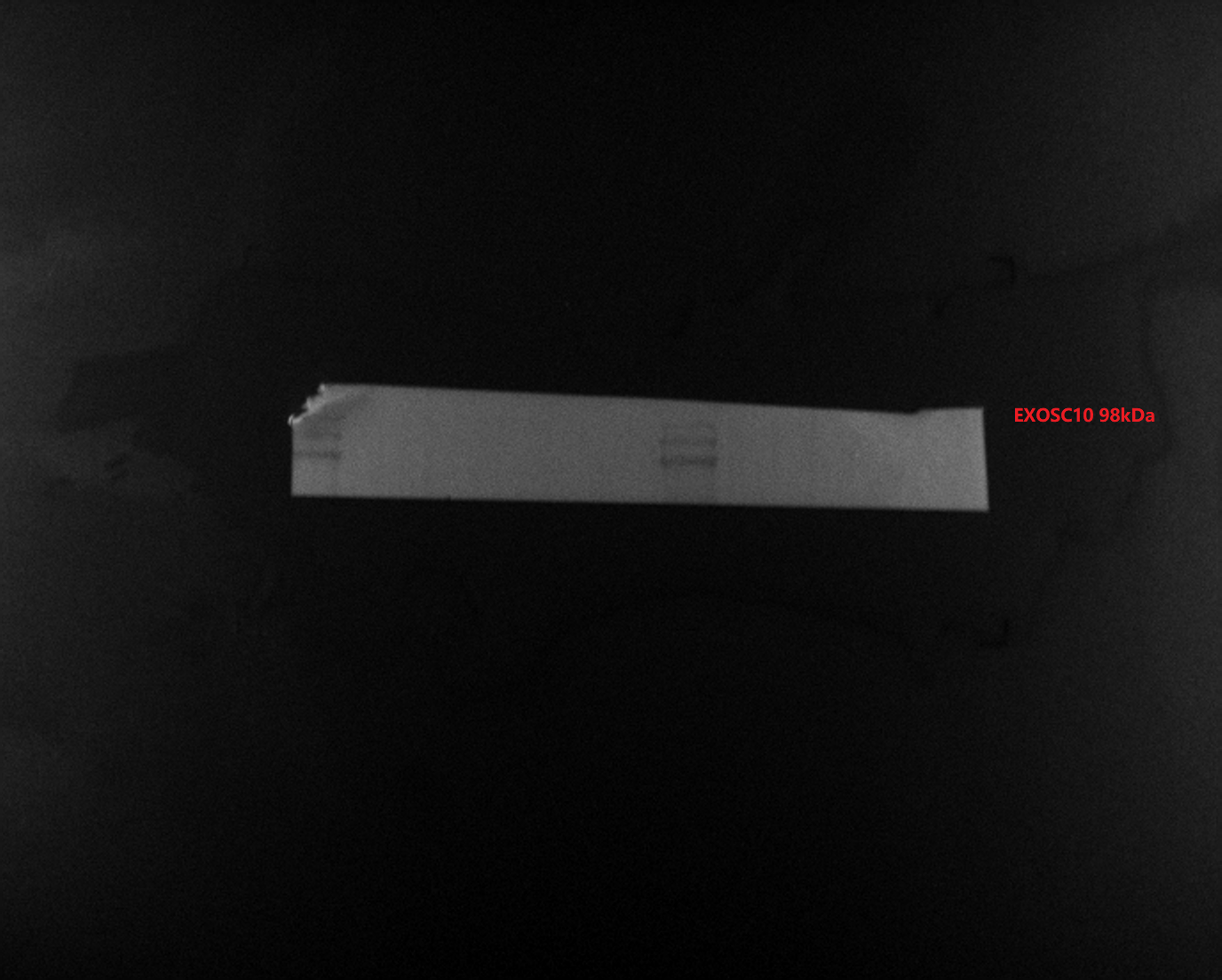

Supplement: Supplemental Information 6 [file peerj-11-15860-s006.zip › raw data-western blot/Western Blots images/Western Blot/Original Image for Fig 8A/EXOSC10-original drawing.tif]

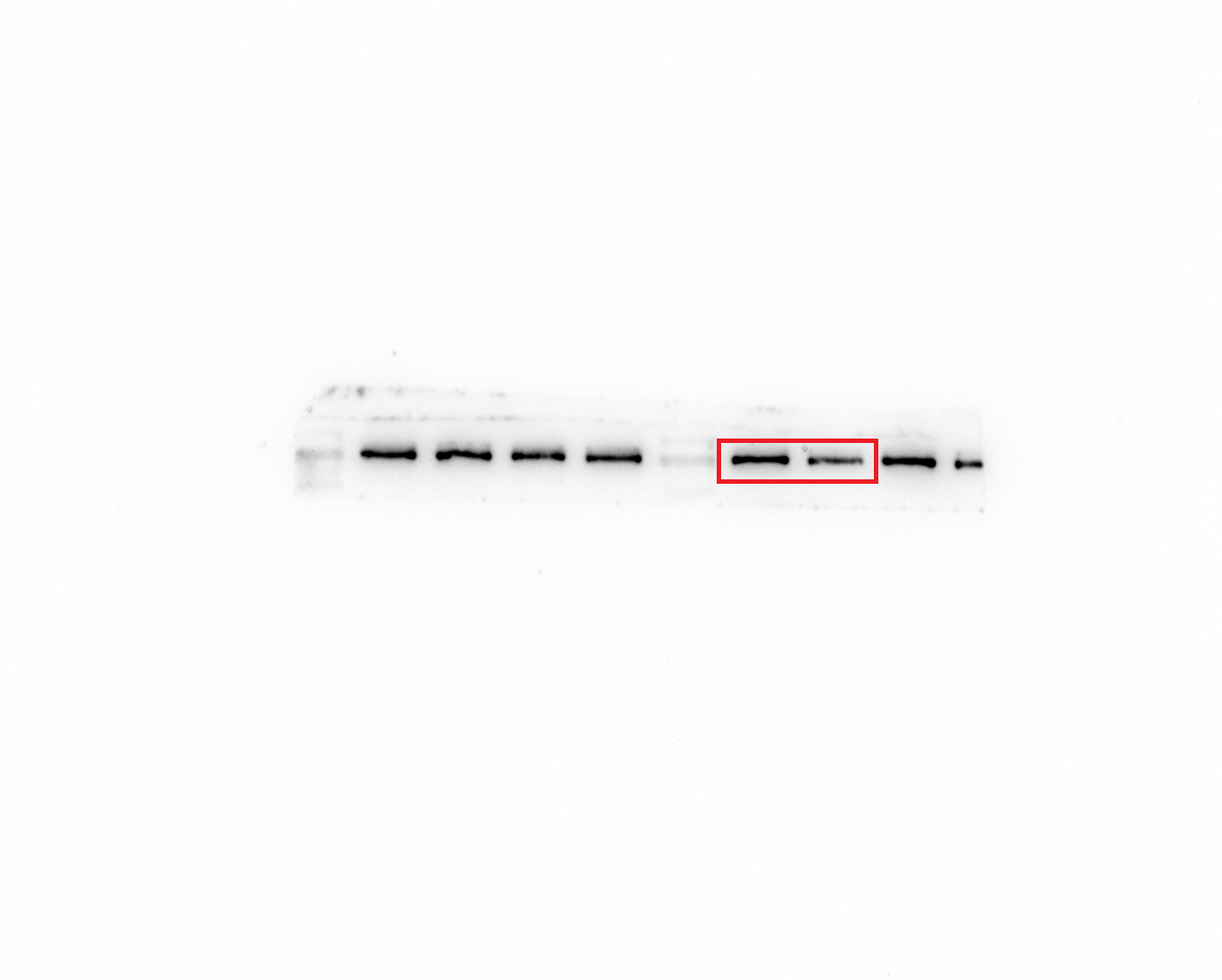

Supplement: Supplemental Information 6 [file peerj-11-15860-s006.zip › raw data-western blot/Western Blots images/Western Blot/Original Image for Fig 8A/EXOSC10.tif]

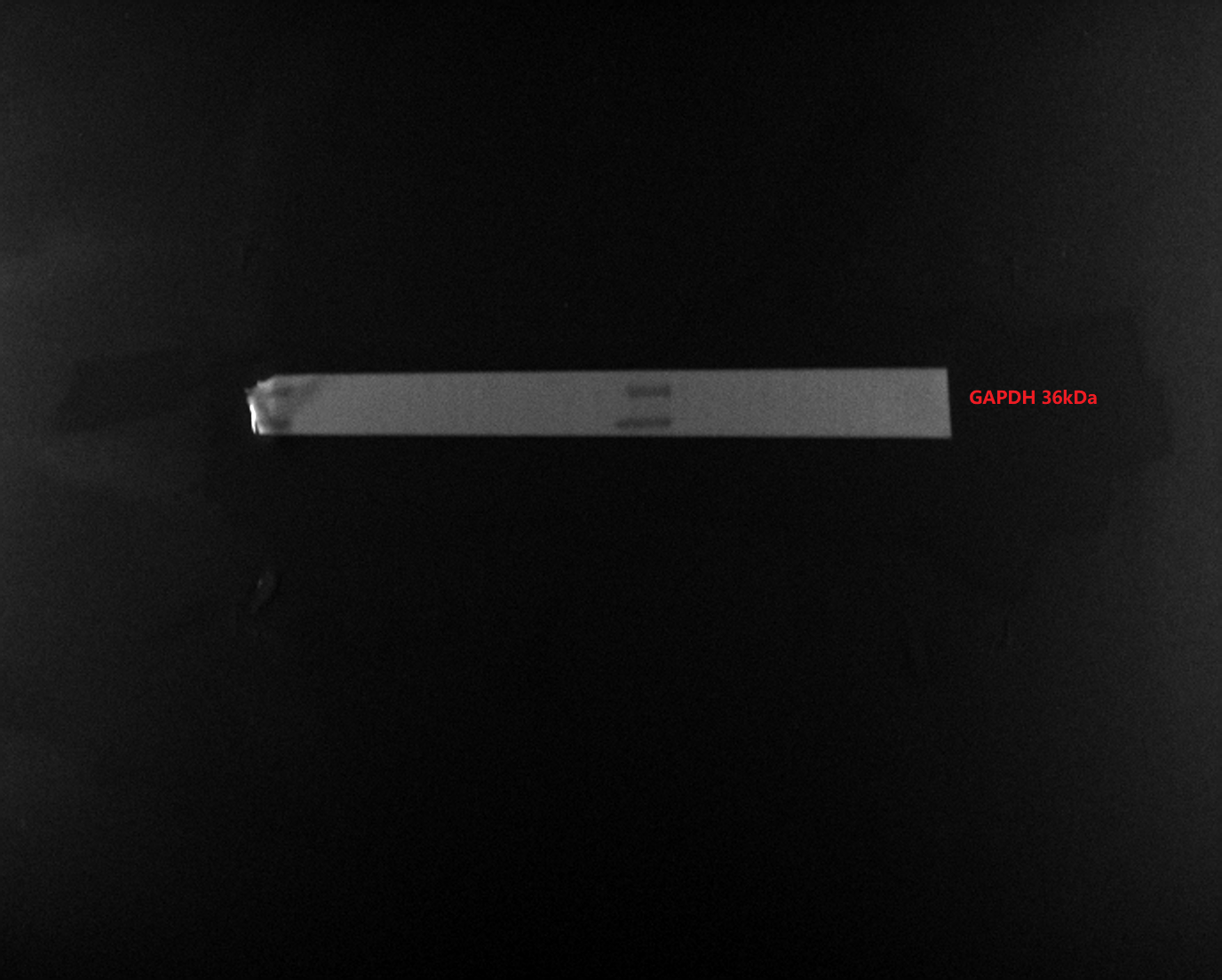

Supplement: Supplemental Information 6 [file peerj-11-15860-s006.zip › raw data-western blot/Western Blots images/Western Blot/Original Image for Fig 8A/GAPDH-original drawing.tif]

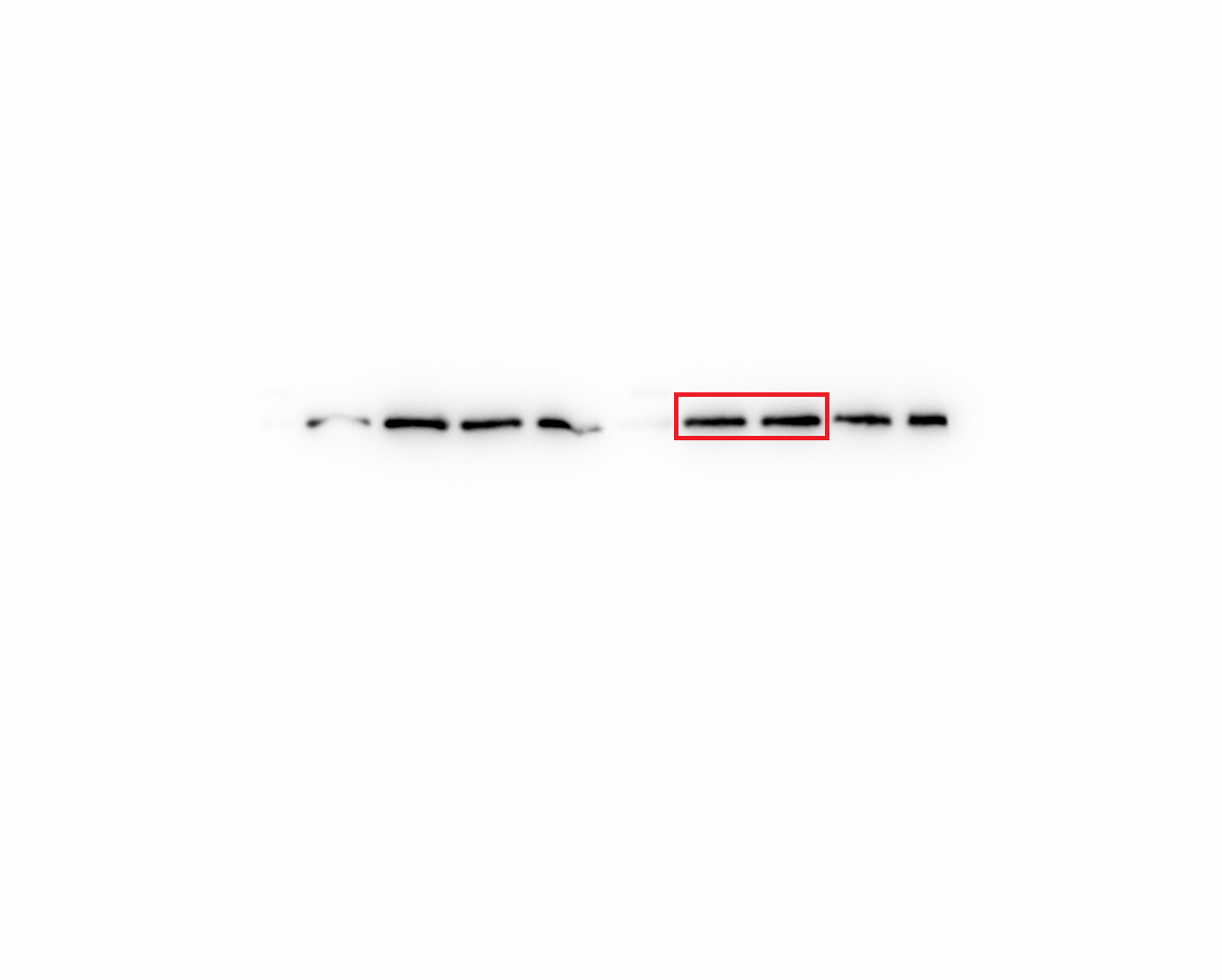

Supplement: Supplemental Information 6 [file peerj-11-15860-s006.zip › raw data-western blot/Western Blots images/Western Blot/Original Image for Fig 8A/GAPDH.tif]

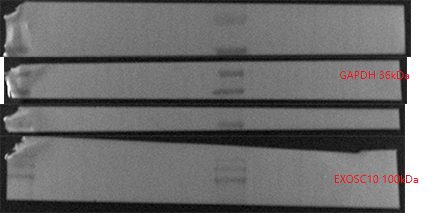

Supplement: Supplemental Information 6 [file peerj-11-15860-s006.zip › raw data-western blot/Western Blots images/Western Blot/Original Image for Fig 8A/Intact membrane.tif]
